# Metaphor—A workflow for streamlined assembly and binning of metagenomes

**DOI:** 10.1093/gigascience/giad055

**Published:** 2023-07-31

**Authors:** Vinícius W Salazar, Babak Shaban, Maria del Mar Quiroga, Robert Turnbull, Edoardo Tescari, Vanessa Rossetto Marcelino, Heroen Verbruggen, Kim-Anh Lê Cao

**Affiliations:** Melbourne Integrative Genomics, School of Mathematics & Statistics, University of Melbourne, Parkville, VIC 3052, Victoria, Australia; Melbourne Data Analytics Platform (MDAP), University of Melbourne, Carlton, VIC 3053, Victoria, Australia; Melbourne Data Analytics Platform (MDAP), University of Melbourne, Carlton, VIC 3053, Victoria, Australia; Melbourne Data Analytics Platform (MDAP), University of Melbourne, Carlton, VIC 3053, Victoria, Australia; Melbourne Data Analytics Platform (MDAP), University of Melbourne, Carlton, VIC 3053, Victoria, Australia; Department of Molecular and Translational Sciences, Monash University, Clayton, VIC 3168, Victoria, Australia; Centre for Innate Immunity and Infectious Diseases, Hudson Institute of Medical Research, Clayton, VIC 3168, Victoria, Australia; School of BioSciences, University of Melbourne, Parkville, VIC 3052, Victoria, Australia; Department of Microbiology and Immunology, The University of Melbourne at the Peter Doherty Institute for Infection and Immunity, Parkville, VIC 3052, Victoria, Australia; School of BioSciences, University of Melbourne, Parkville, VIC 3052, Victoria, Australia; Melbourne Integrative Genomics, School of Mathematics & Statistics, University of Melbourne, Parkville, VIC 3052, Victoria, Australia

**Keywords:** bioinformatics, pipeline, MAGs, Snakemake, high-throughput sequencing, microbial genomics

## Abstract

Recent advances in bioinformatics and high-throughput sequencing have enabled the large-scale recovery of genomes from metagenomes. This has the potential to bring important insights as researchers can bypass cultivation and analyze genomes sourced directly from environmental samples. There are, however, technical challenges associated with this process, most notably the complexity of computational workflows required to process metagenomic data, which include dozens of bioinformatics software tools, each with their own set of customizable parameters that affect the final output of the workflow. At the core of these workflows are the processes of assembly—combining the short-input reads into longer, contiguous fragments (contigs)—and binning, clustering these contigs into individual genome bins. The limitations of assembly and binning algorithms also pose different challenges depending on the selected strategy to execute them. Both of these processes can be done for each sample separately or by pooling together multiple samples to leverage information from a combination of samples. Here we present Metaphor, a fully automated workflow for genome-resolved metagenomics (GRM). Metaphor differs from existing GRM workflows by offering flexible approaches for the assembly and binning of the input data and by combining multiple binning algorithms with a bin refinement step to achieve high-quality genome bins. Moreover, Metaphor generates reports to evaluate the performance of the workflow. We showcase the functionality of Metaphor on different synthetic datasets and the impact of available assembly and binning strategies on the final results.

## Introduction

Genome-resolved metagenomics (GRM) is a set of techniques for the recovery of genomes from high-throughput sequencing data. Applications of GRM have led to unprecedented insight into microbial diversity, ecology, and evolution, due to the recovery of (mostly uncultivated) metagenome-assembled genomes (MAGs) [1–4]. MAGs are essentially “bins” of contigs that are clustered together based on differential coverage and sequence composition; a bin is considered a MAG when it displays a high degree of completeness and a low degree of redundancy/contamination, which is usually calculated through the presence of marker genes in the bin. Advances in GRM have consistently improved the quality of recovered MAGs, and large-scale studies reconstructing and analyzing thousands of MAGs have become prominent in microbiology research. Even with the inherent biases that accompany the generation of MAGs, it is evident that the benefits outweigh the risks, and researchers are increasingly in need of automated data processing methods for assembling and binning metagenomes [5]. Data pipelines that perform such experiments are inherently complex, have high computing cost, use heterogeneous data sources, have dozens of customizable parameters, and depend on several specialized bioinformatics software [6, 7].

An additional domain-specific challenge for GRM studies is the strategy used for assembling and binning each sequenced sample. Data (raw reads generated by the sequencer) originating from multiple samples may be assembled separately or pooled together, depending whether they come from the same population, specimen, or environment. This results in either a set of contigs for each sample or a “coassembly” of the pooled samples. Similarly, in the metagenome binning step, where contigs are clustered into genome bins, one may do this individually for each set of assembled contigs or by pooling together contigs from multiple samples and then mapping each individual sample to this catalog of contigs (“cobinning”) [8]. The latter approach allows binning algorithms to account for differential coverage of contigs across samples, enriching the information available for clustering. The chosen strategy for assembly and binning may have important consequences for the final results (i.e., the quality of the assembly and of the recovered bins) [8]. It is hypothesized that pooled assembly and binning may lead to improved results when analyzing communities with high genetic diversity and to poorer results when there is a high level of intraspecies/strain-level diversity [9],

Here we present Metaphor, an automated and flexible workflow for the assembly and binning of metagenomes, which recovers prokaryotic genomes from metagenomes efficiently and with high sensitivity, and it provides taxonomic and functional abundance data for quantitative metagenome analyses. Our software advances existing metagenomic pipelines by combining 2 core features: the usage of multiple binning software, along with a binning refinement step, and the possibility of defining groups for assembly and binning of samples. This effectively allows scaling Metaphor to process multiple datasets in a single execution, performing assembly and binning in separate batches for each dataset, and avoiding the need for repeated executions with different input datasets. The workflow includes native functionality for downstream integration with omics statistical toolkits [10, 11], so that abundance data can be easily imported into these tools, and with the Anvi’o [12] platform, which allows importing the collections of bins generated by Metaphor along with contig coverage data. Metaphor generates detailed performance metrics at the end of each module of the workflow to provide users with a high-level summary of their analysis, and it has been designed to be user-friendly, portable, and flexible, as users can choose between different strategies for assembly and binning. We demonstrate its functionality using different synthetic datasets and discuss how these different strategies can impact data analyses in terms of quality of the resulting assembly and genome bins.

## Design and Implementation

Metaphor stands out from existing GRM pipelines by offering flexible options for assembly and binning combined with multiple binning software and a binning refinement step. See Table 1 for a comparison of Metaphor’s features with other state-of-the-art GRM workflows. The workflow is implemented with Snakemake [13], a widely used scientific workflow management system. In each module, computing steps (called “rules” by Snakemake) consist of both third-party bioinformatics software [14–28] and custom scripts that connect different parts of the workflow, listed in Table 2.

**Table 1:** Comparison of features between Metaphor and state-of-the-art GRM workflows as listed by [29]. Data adapted to include Metaphor

Features	Metaphor v1.7.7	ATLAS [30]	MetaWRAP [31]	nf-core/mag [32]	MAGNETO [29]
Preprocessing					
Reads trimming	✓	✓	✓	✓	✓
Contamination	✓	✓	✓	✓	✓
Assembly					
Coassembly possible	✓		✓	✓	✓
Coassembly by groups	✓				
Compute sets to coassemble				✓	
Assembly evaluation	✓				
Binning					
Cobinning possible	✓		✓	✓	✓
Multiple binning software	✓	✓	✓		
Bin refinement	✓	✓	✓		
Bin reassembly		✓	✓		
Postprocessing					
MAGs quality check	✓	✓	✓	✓	✓
Dereplication step	✓	✓	✓	✓	✓
Genome annotation	✓	✓	✓	✓	✓
Gene catalog	✓			✓	✓
HTML report	✓	✓		✓	✓
Reproducibility					
Workflow management	✓	✓		✓	✓
Packages management	✓	✓		✓	✓

**Table 2:** Modules, steps, and software used in Metaphor

Module	Step	Software
Quality control (QC)	Trim adapters and filter low-quality reads	fastp [14]
	Generate QC reports	FastQC [15]
	Combine QC reports	MultiQC [16]
Assembly	Assemble filtered and merged reads into contigs	MegaHit [17]
	Perform assembly evaluation	MetaQUAST [18]
	Assemble report and plots	Metaphor script*
Mapping	Map reads	MiniMap2 [19]
	Sort and index mapped reads	Samtools [20]
Annotation	Predict coding sequences from contigs	Prodigal[21]
	Annotate coding sequences	Diamond, NCBI COG [22, 23]
	Annotate MAGs	Prokka [24]
	Annotate report and plots	Metaphor script*
Binning	Cluster contigs into bins	VAMB [25]
	Cluster contigs into bins	MetaBAT2 [26]
	Cluster contigs into bins	CONCOCT [27]
	Dereplicate and score bins	DAS Tool [28]
	Binning report and plots	Metaphor script*
Postprocessing	Concatenate benchmarks	Metaphor script*
	Plot benchmarks	Metaphor script*

* External libraries used in Metaphor scripts: [33–35].

**Figure 1:**
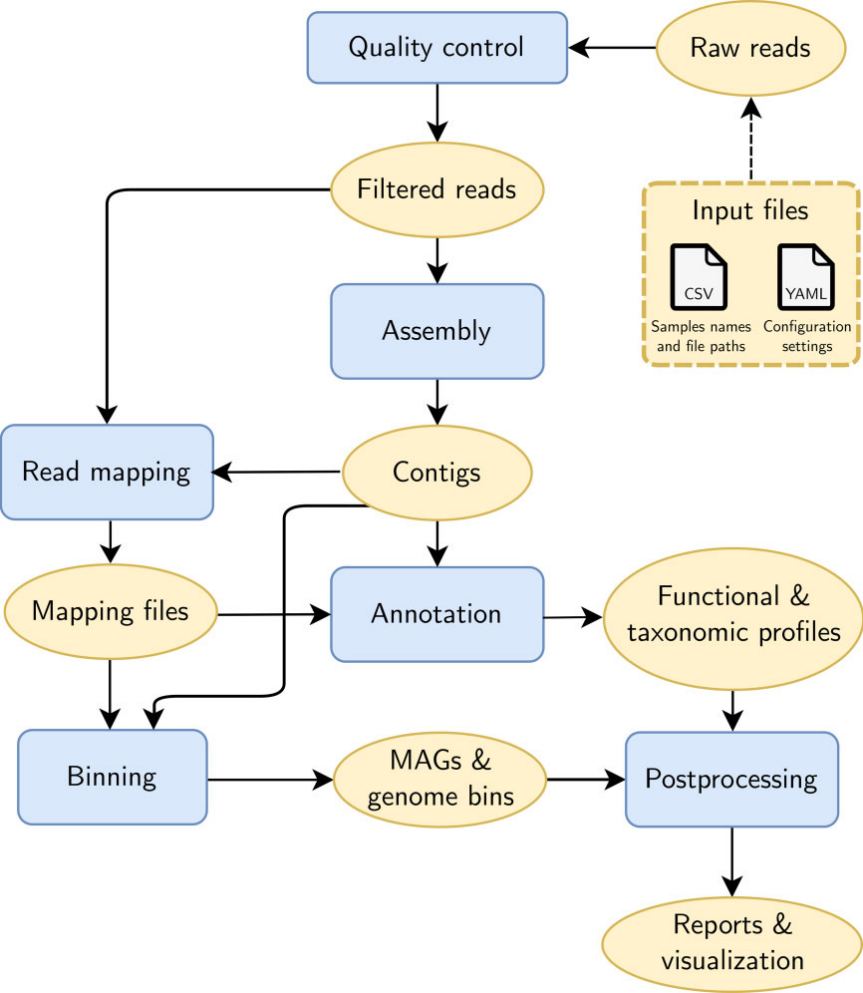
Simplified workflow diagram. Workflow modules are represented by rectangular blue shapes and data files are represented by oval yellow shapes, except for entrypoint files shown in a dashed yellow rectangle. Arrows indicate input and output of data between modules.

The workflow consists of 6 modules: quality control (QC), assembly, annotation, mapping, binning, and postprocessing. In the QC module, raw sequencing reads are filtered and trimmed. Metagenomic assembly is then performed. Coding sequences are predicted from the assembled contigs and used for functional and taxonomic annotation. The quality-filtered reads are mapped against the contigs, generating coverage statistics employed by the binning algorithms. After binning is complete, bins are refined and dereplicated. Lastly, the postprocessing module renders runtime and memory usage metrics and generates an HTML report. A simplified version of the flow of data between the different modules of the workflow is show in Fig. 1.

The choice of bioinformatics tools was informed by the results of the Second Critical Assessment for Metagenome Interpretation (CAMI II) [8, 36], striving for the maximum trade-off between performance, efficiency, and software sustainability. Although the latter is a subjective factor, selecting and streamlining dependencies with regard to code quality, maintenance, and community support is a critical factor when maintaining complex bioinformatics pipelines [6, 37]. Each third-party software (along with its version) is defined in an individual requirements file that is used by Snakemake to create a virtual environment and run that particular step. To facilitate citing these tools, Metaphor packages a bibs/ directory containing all citations in the Bibtext format.

The workflow takes 2 files as input: a tab-delimited file containing sample names and file paths to the raw reads and a configuration file in the YAML format, which will set the workflow parameters (see Fig. 1). These files can be automatically generated by Metaphor and edited by the user or created from scratch. The output of Metaphor consists of a directory for each module, further subdivided into the rules within each module. This is described in detail in the documentation [38].

### Assessment on CAMI II synthetic datasets

To demonstrate the functionality of Metaphor, we analyzed datasets from CAMI II [8], described in Table 3. All datasets consist of short and long reads generated by simulation of collections of reference genomes [39]. Only short reads were used for each dataset, as Metaphor does not yet support long reads. Specifically, we used the Marine metagenome dataset (identified as “marmg”), the Strain Madness dataset (identified as “strmg”), and the Human Microbiome dataset, which consists of 5 sets of samples, each corresponding to a different sampling location in the human body, which were treated as distinct datasets [3]. The following strategies were employed for each dataset: single assembly, single binning (SASB), where each sample is individually assembled and binned; single assembly, cobinning (SACB), where each sample is assembled individually and then binned with other samples from the same dataset; and coassembly, cobinning (CACB), where all samples from the dataset are assembled and binned together. Table 4 illustrates how this works in practice, in terms of generated output files. Metaphor allows defining multiple groups for coassembly or cobinning to analyze multiple independent datasets with a single execution.

**Table 3:** Datasets from CAMI II used to assess the workflow. Columns show the number of samples and size in gigabytes of each dataset, along with the amount of reference genomes used to generate the dataset

Dataset	Identifier	No. of samples	Size (GB)	No. reference genomes
Marine	marmg	10	50	622
Strain Madness	strmg	100	200	408
Human Airways	h_airways	10	44	1,394
Human Genital	h_urogenital	9	39	1,394
Human Gut	h_gastrointestinal	10	44	1,057
Human Oral	h_oral	10	43	1,057
Human Skin	h_skin	10	44	1,394

**Table 4:** Output files for each strategy. If only 1 dataset/group is being analyzed, assembly and binning results are named as “Coassembly” and “Cobinning,” respectively. If multiple datasets/groups are used, the results are named according to the group/dataset’s name

Strategy	Description	Reads files	Assemblies	Bins
SASB	Single assembly, single binning	Sample_0.fastq	Sample_0_contigs.fasta	Sample_0_bins/
		Sample_1.fastq	Sample_1_contigs.fasta	Sample_1_bins/
		Sample_2.fastq	Sample_2_contigs.fasta	Sample_2_bins/
SACB	Single assembly, cobinning	Sample_0.fastq	Sample_0_contigs.fasta	Cobinning_bins/
		Sample_1.fastq	Sample_1_contigs.fasta	
		Sample_2.fastq	Sample_2_contigs.fasta	
CACB	Coassembly, cobinning	Sample_0.fastq	Coassembly_contigs.fasta	Cobinning_bins/
		Sample_1.fastq		
		Sample_2.fastq		

In order to assess the effect of different assembly strategies, we used MetaQUAST [18] to compare the assemblies generated by the workflow with the collections of reference genomes. For the different binning strategies, we compared metrics obtained from DAS Tool, the software used for dereplicating and evaluating genome bins, after a second round of dereplication with dRep [40]. This is because data generated with the SASB strategy will likely result in redundant bins, as for that strategy, there is no dereplication between samples, and since samples within a dataset have similar composition, it is likely that a genome bin can be generated repeatedly by different samples. dRep performs dereplication based on the average nucleotide identity between genomes, a metric that has been consistently used as a proxy to differentiate taxonomy at the species and strain levels [41]. dRep was run with default clustering parameters and without any length, completeness, or contamination cutoffs. We used Spartan [42], the High Performance Computing system at the University of Melbourne, to run the pipeline. Jobs were dispatched to nodes with the SLURM scheduler, using up to 64 processors and 300 GB RAM per node.

## Results and Discussion

After running Metaphor on the CAMI II Marine, Strain Madness, and Human Microbiome datasets, we illustrate the different outputs generated by the workflow and compare the effects of different assembly and binning strategies on workflow performance.

### Reconstruction of metagenome-assembled genomes

**Figure 2:**
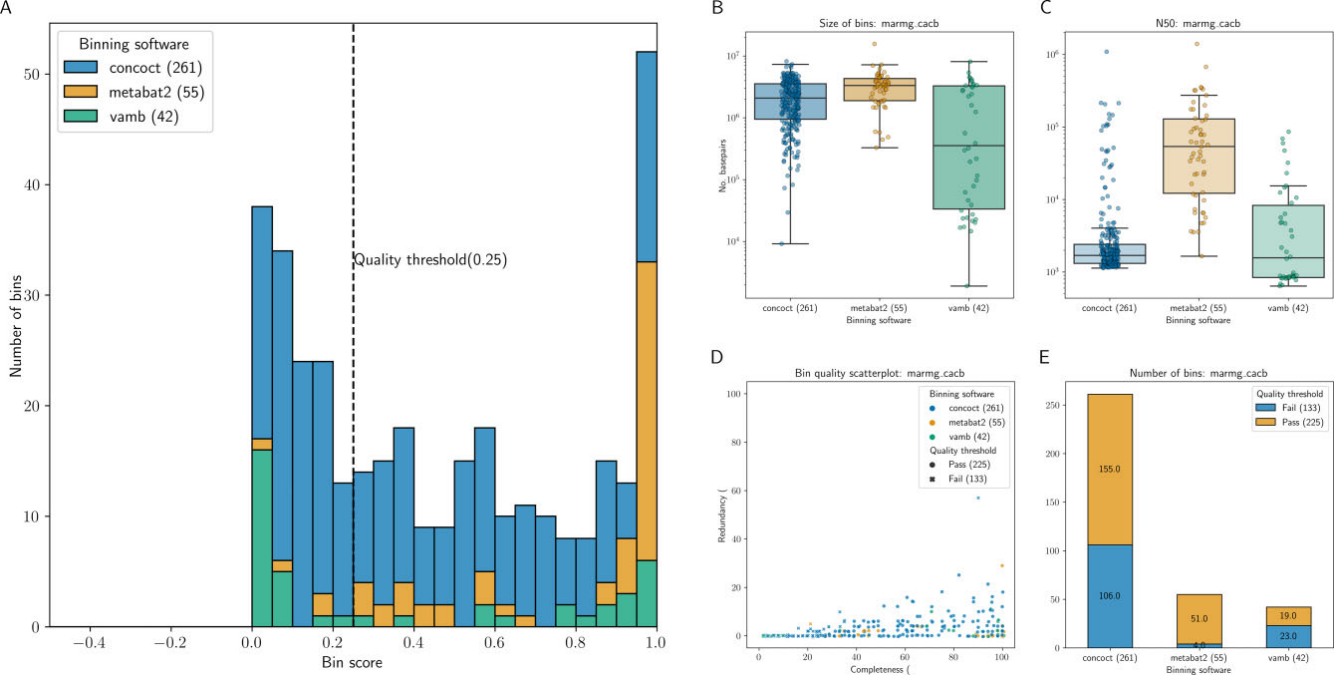
Binning report generated by Metaphor for the CAMI II Marine metagenome dataset processed with the CACB (coassembly, cobinning) setting. (A) A stacked histogram of the distribution of bin scores, with the defined quality threshold highlighted as a dashed line. (B) The size (in base pairs) and (C) N50 of bins. The y-axis is in log-scale. (D) Scatterplot of completeness and redundancy for each bin. Colors indicate the tool used to generate the bin, and the symbols indicate whether that bin passed or failed the bin score quality threshold (corresponding to the same value in the dashed line of A). (E) The number of bins that passed or failed the quality threshold for each binning tool.

Metaphor produces genome bins generated with 3 tools—Vamb, MetaBAT2, and CONCOCT [25–27]—that are refined with the DAS Tool [28]. The DAS Tool performs bin refinement through a “dereplication, aggregation, and scoring” process, in which candidate bins are initially scored based on the presence/absence of single-copy marker genes (SCGs, which are a proxy for bin completeness). Redundant candidate bin sets are then aggregated, and an iterative scoring process is performed, so only the best-quality, nonredundant bins remain; the bin score (*S_b_*) increases with the number of SCGs and decreases with duplicate SCGs per bin. Please refer to [28] for an overview of the DAS algorithm and the formula to determine the bin score. The input for each binning tool differs slightly, but they all rely on the catalog of contigs obtained from the assembly and the coverage files obtained from the read mapping module (see Fig. 1). A report is generated for each of the binning groups (only 1 is generated if cobinning is performed), which highlights 3 key metrics: completeness, redundancy, and bin score. The first 2 metrics are calculated by the presence/absence of single-copy genes, and the latter is a function of the former two. Plots generated by an example report are shown in Fig. 2. It is possible to compare the performance of the different binning software and obtain the proportion of bins above a specified particular quality threshold based on the bin score. The source table for the report is provided, so that users can generate custom reports and inspect specific individual bins. Bins that pass the quality threshold are stored in individual FASTA files, so they can easily be used for downstream analyses with tools such as CheckM or GTDB-Tk [43, 44]. We chose not to include these software in the workflow as they rely on fairly large reference databases and/or contain several different steps that are dependent on third-party software, which would affect Metaphor’s portability. Bin collections generated with Metaphor can be imported into the Anvi’o along with coverage data (BAM files), so users can use the interactive interface of Anvi’o to examine the bins.

### Contig-level taxonomic and functional profiling

**Figure 3:**
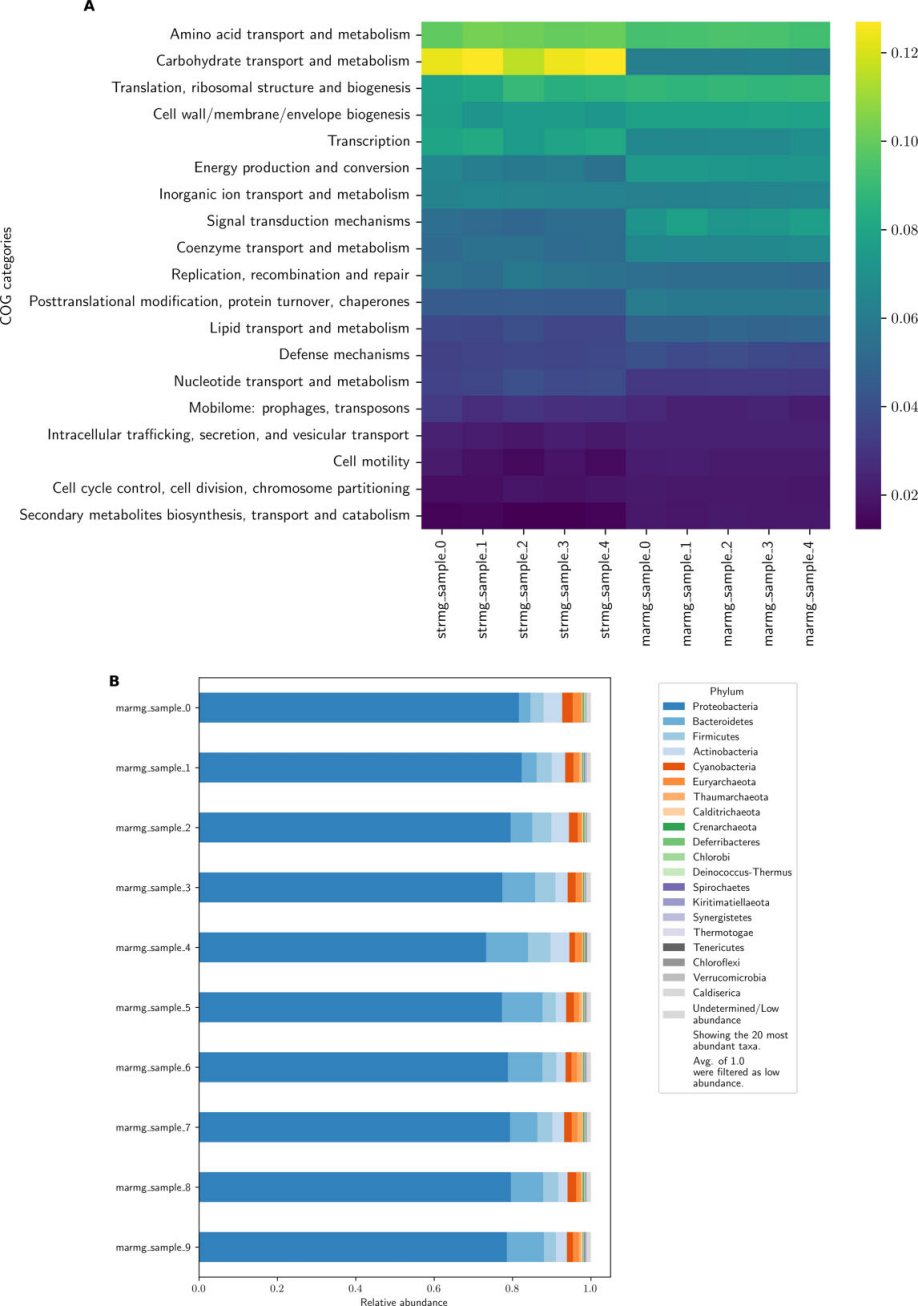
Annotation plots generated by Metaphor on the Strain Madness (“strmg”) and the Marine (“marmg”) datasets. (A) The functional profile as a heatmap of the relative abundance of functional COG categories (y-axis) across samples (x-axis) for 5 samples from the Strain Madness and Marine datasets. (B) The taxonomic profile of the Marine dataset as a stacked barplot of relative abundance of taxa. In this case, the phylum rank was used, but Metaphor generates this for the most common taxonomic ranks (phylum, class, order, family, genus, species). The number of abundant taxa can be easily adjusted in the workflow settings. For both taxonomic and functional profiles, abundance of each feature is calculated from coverage values for each gene.

To facilitate quantitative metagenomics applications, Metaphor’s annotation module generates contig-level functional and taxonomic profiles based on the NCBI COG database [23]. These are obtained by predicting coding sequences with Prodigal and then aligning the resulting amino acid files with Diamond [21, 22] in the “iterative” mode. This setting performs repeated rounds of alignment, with an increasing degree of sensitivity when no hits are detected in the previous round. Abundances for each feature are calculated based on the coverage of all coding sequences that align to that feature. Figure 3 illustrates the profile visualizations offered by Metaphor: a heatmap of COG categories for the functional profile and a stacked barplot for the most abundant taxa (for the latter, 1 plot is generated for each taxonomic rank). The annotation module outputs count tables with both absolute and relative abundance values of taxa and functional categories and may be directly imported by downstream statistical toolkits such as MixOmics or PhyloSeq [10, 11].

### Quality control and performance metrics

**Figure 4:**
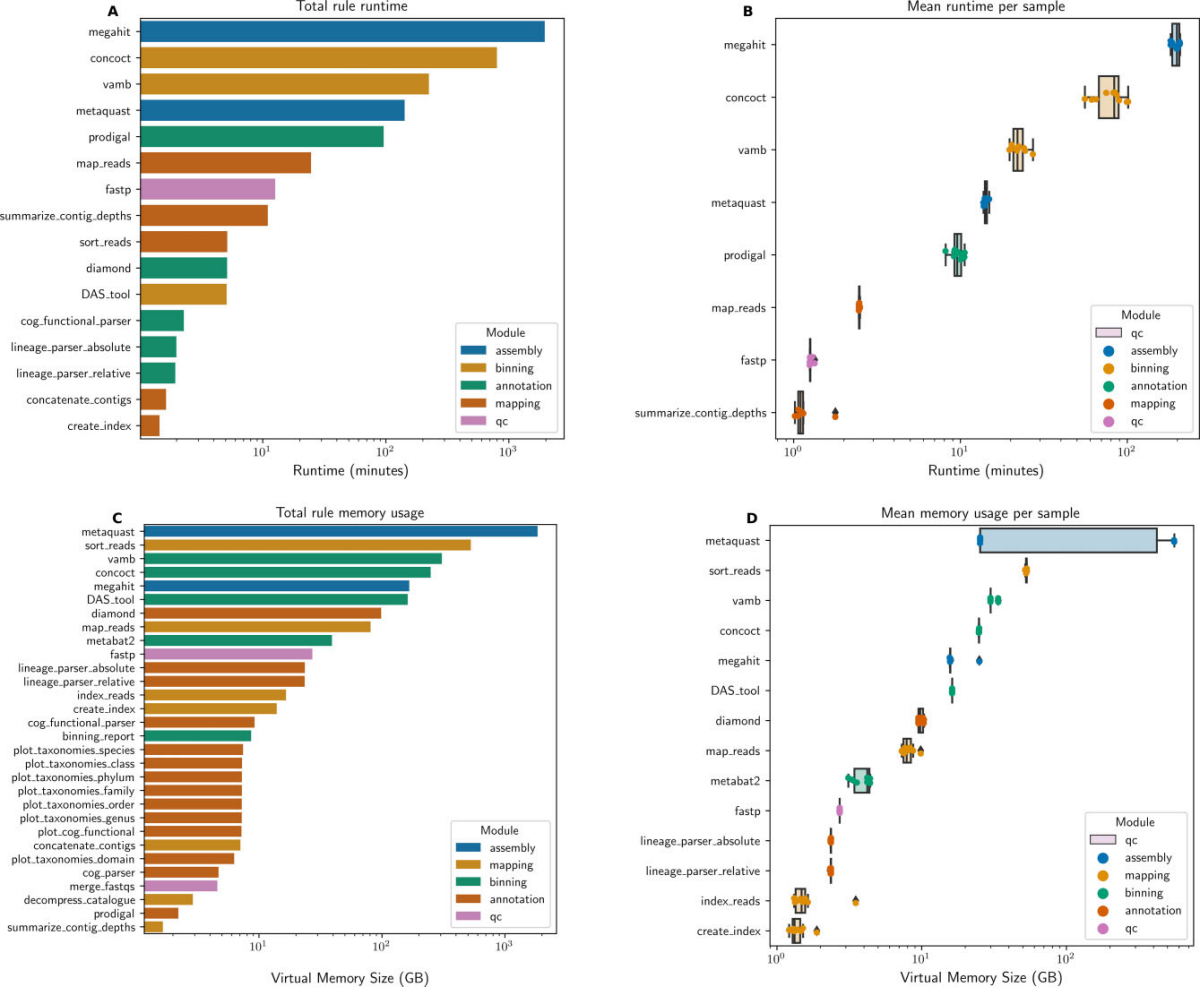
Performance metrics report generated by Metaphor on the Marine dataset processed with the SASB strategy. Total runtime per rule (A), mean runtime per sample (B), total memory usage per rule (C), and mean memory usage per sample (D). The x-axis is in log format. Cutoffs are applied to omit rules with short runtime or low memory usage. Colors indicate the workflow module of each rule.

Additional outputs produced by Metaphor include the quality control reports from the fastp and FastQC tools, with a summary of FastQC outputs being produced by MultiQC [14–16]. A simple report is produced by the assembly module with sequence statistics of the assembled contigs (e.g., N50, number of contigs, total and mean length of contigs), and performance metrics. At the end of the workflow execution, the postprocessing module generates figures obtained from the “benchmark” files provided by Snakemake. These files contain process information such as runtime and memory consumption. Metaphor plots these metrics in 2 ways: total per rule and per-sample mean (Fig. 4) as some rules run only once across all samples, while other rules run per sample. These plots help identify computational bottlenecks and assess whether computing resources are adequate.

### Assembly and binning strategies

The effects of distinct assembly and binning strategies on the final output of metagenomic workflows are highly dependent on the data source and research context [8]. As such, the choice of individual or group assembly and binning can only be assessed a posteriori. We compared 3 different strategies: SASB, SACB, and CACB; see Tables 3 and 4 and “Assessment on CAMI II synthetic datasets” section for details. For assembly, we used the 5 different groups in the Human Microbiome dataset along with the Strain Madness and Marine datasets. We only used the latter 2 datasets for the binning assessment.

**Figure 5:**
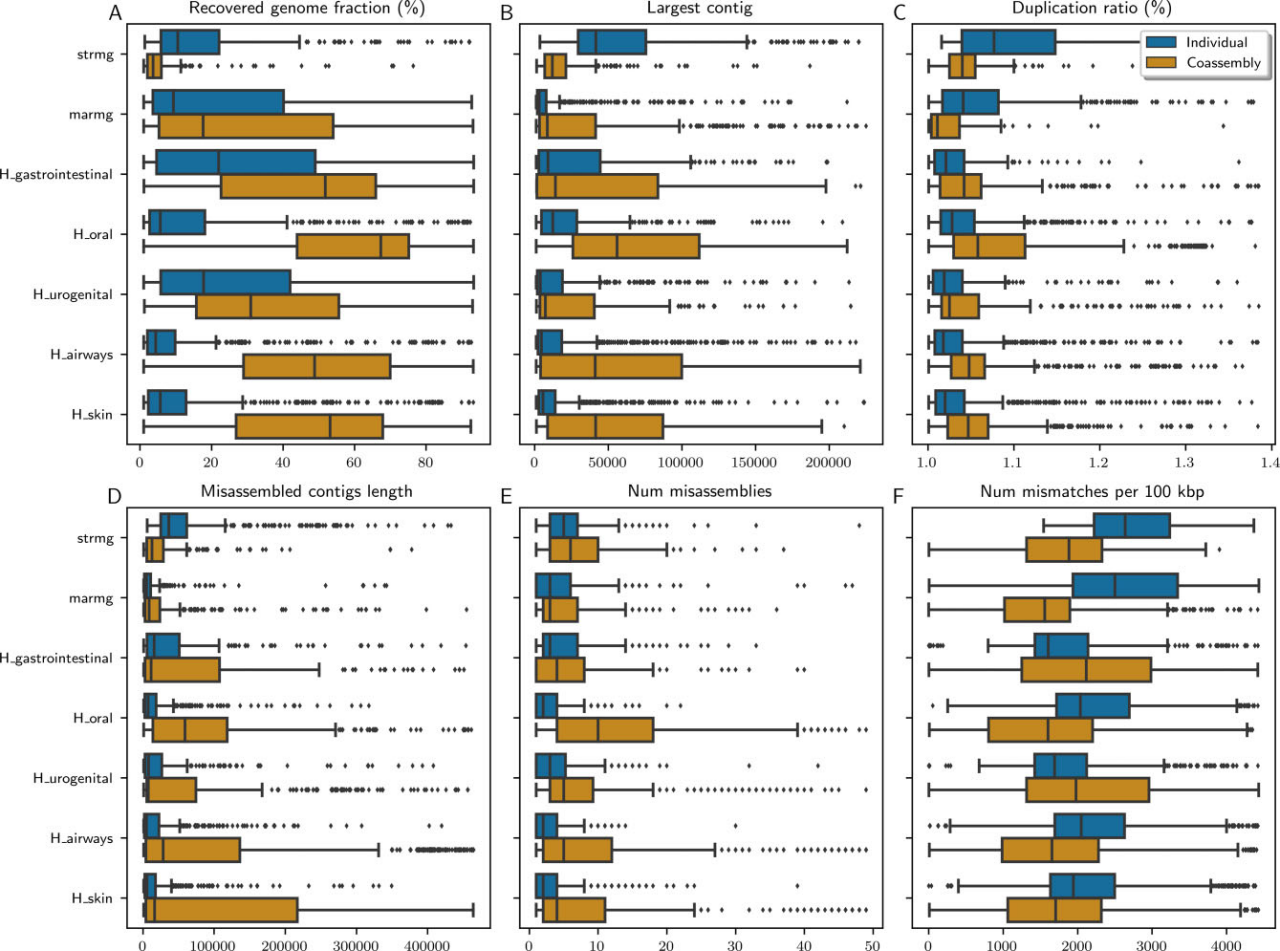
Differences between assembly strategies for each dataset. Each data point corresponds to a reference genome evaluated with the MetaQUAST tool. Data points above the 98th percentile were classified as outliers and removed from the figure to improve visualization. See Supplementary Fig. S1 for the full data. The title at the top of each panel indicates the plotted metric. Panels A and C show percentages along the x-axis, while the remainder show absolute values.

We used 6 metrics to evaluate assembly performance: percentage of recovered genome fraction, size of the largest contig, duplication ratio, length of misassembled contigs, number of misassemblies, and number of mismatches per 100,000 base pairs. High values for the first 2 metrics and low values for the last 4 indicate better performance. We observed a general trade-off between assembly completeness (represented by the first 2 metrics) and the number of errors in the assembly (represented by the last 4 metrics), shown in Fig. 5 (Supplementary Fig. S1). In most datasets, assemblies were more complete and contiguous, albeit with more errors when the coassembly strategy was used. The exception was the Strain Madness (“strmg”) dataset, for which the individual assembly was more complete and contiguous, albeit with more errors. This may be attributed to the high degree of strain/intraspecies diversity in that dataset [8]. A high degree of similarity between the related genomes likely confounds assembly algorithms, and pooling samples together may aggravate this effect [5].

To evaluate differences between binning strategies, we compared the number and quality of bins after refinement with the DAS Tool. Bins generated with each approach were further dereplicated with dRep [40]. This is because the SASB strategy generates a set of bins for each sample, and datasets with similar composition will likely generate redundant bins, as there is no dereplication of bins between samples. Results varied significantly between the Marine and Strain Madness datasets. In both datasets, the mean bin score was the highest for the CACB strategy (Supplementary Fig. S2). However, in the Strain Madness dataset, CACB produced a significantly lower number of bins (33 compared with 259 and 215 generated with SASB and SACB, respectively), which did not occur in the Marine dataset. The performance of each binning tool is also variable between strategies and conditional on the characteristics of the original dataset, with no clear “winner,” and each tool favoring particular performance metrics, in agreement with results from the CAMI II challenge [8]. Tools like DAS Tool attempt to conciliate the output of multiple binning algorithms to generate a consensus output that theoretically outperforms each individual algorithm.

**Figure 6:**
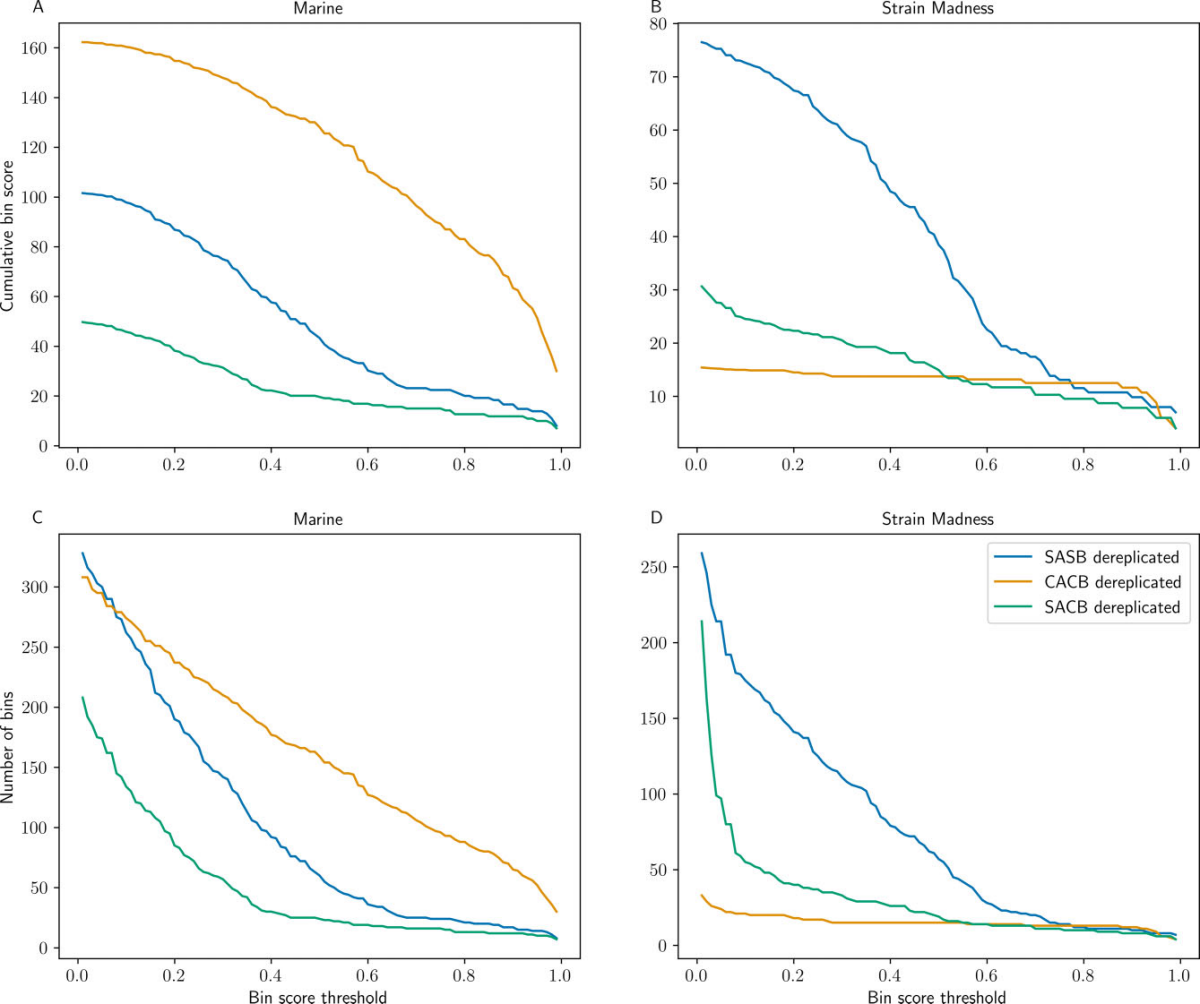
Cumulative bin score and number of bins between binning strategies for the Marine and Strain Madness datasets. Lines show the cumulative bin score (A, B) and number of bins (C, D) along the y-axis, for bins above a certain score threshold (x-axis). Left column shows Marine dataset, and right column shows Strain Madness dataset.

Since the binning performance is assessed as a proxy of the combination of quantity and quality of generated bins, rather than only one metric or the other, we calculated the cumulative bin score (the sum of scores of all bins) and the number of bins above an increasing score threshold, shown in Fig. 6. The higher the threshold, the more significant the differences between the cumulative scores, as only bins with the highest quality compose the score. For the Marine dataset, we observed a higher score and a larger number of bins in the CACB strategy and the exact opposite in the Strain Madness dataset. In both datasets, there was a clear difference between SASB before dereplication and the other strategies, confirming that several highly similar samples produce redundant bins. That difference was also present in the SACB strategy, albeit not so pronounced (see Supplementary Figs. S2, S3 and S4 for the comparison of dereplicated and non-dereplicated data). This suggests that for both of these strategies, further dereplication is recommended [5]. Although the Strain Madness dataset shows fewer bins generated with CACB, a summary of the bins recovered with that dataset is displayed in Supplementary Table S1. The cumulative bin score for that strategy remained similar to SACB and SASB above the 0.8 score threshold, since there are fewer bins with a score lower than that. In that same dataset, SASB showed the best performance, although differences were small above the 0.8 threshold. In the Marine dataset, there were more pronounced differences between strategies. CACB produced the larger quantity and higher cumulative score of bins, followed by SASB and SACB.

In summary, our results indicate that, for most metagenomic analysis scenarios, coassembly followed by cobinning is recommended, assuming that samples are sourced from a similar environment or population. The exception to this is when when there is a high level of intraspecies/strain-level diversity across samples, like in the Strain Madness dataset. In that scenario, single assembly followed by single binning is preferred, followed by dereplication of bins between samples. There is, however, a trade-off between the different approaches, as computational requirements are higher for the pooled strategies. Coassembly resulted in higher genome recovery fractions and larger contigs, although usually at the expense of a higher number of misassemblies and higher duplication ratio. When combining coassembly with cobinning, there is a remarkable improvement in the quantity and quality of bins generated for a diverse dataset (represented by the Marine dataset), whereas the difference was negligible in the Strain Madness dataset. Therefore, when deciding the assembly and binning strategy, it is important to consider the expected strain-level diversity and abundances of each individual genome, as the interaction between these factors is likely to limit the resolution of recovered bins. This is shown in the CAMI II challenge [8] (see Fig. 1G); genomes with low strain diversity (i.e., are less than 95% similar to any other genome) have a higher correlation between sequencing coverage and recovered fraction than common genomes (≥95% similar to other genomes in the sample), although many times, sequencing coverage was not all correlated with genome recovery fraction, especially for smaller bins that represent plasmids or circular elements.

## Availability and Future Directions

Metaphor is available through Bioconda [45], a popular repository of bioinformatics software. It can be installed with a single command from the conda package manager [46] or from source using pip, the Python package manager. The installation of all third-party software used by Metaphor is handled automatically by Snakemake and conda. It can be easily deployed in different computing environments, such as high-performance computing clusters and cloud instances, due to Snakemake’s support of execution profiles. Metaphor is developed with documented best practices in workflow development [6, 47], striving for reproducibility and transparency of its results. Data used for testing Metaphor’s installation (see documentation for details) are available from GitHub at https://github.com/vinisalazar/mg-example-data. These data are a subset of the CAMI I challenge data [36] that are reduced in size in order to run test commands in a reasonable time.

The workflow may be extended to support downstream tools such for genome analysis such as GTDB-Tk, CheckM, and dRep. This may help with further improvement of strain-level resolution in bins; there are a number of strategies for that, such as identification of misassembled contigs or using the assembly graph for variant detection [48, 49]. New functionality may also be added for the identification of eukaryotic and viral contigs; Metaphor would benefit from new third-party software to facilitate the generation of non-prokaryotic bins in the near future. The output of Metaphor’s “annotation” module is suitable for *ad hoc* identification of eukaryotic and viral contigs; after selecting the annotated prokaryotic contigs, it is possible to filter them out, leaving unannotated (putative) eukaryotic and viral contigs. These can then be used as input for a eukaryotic or viral discovery pipeline [50–52], but this process could be further improved by facilitating the use of custom reference databases in the annotation module. This can also be done directly with the output of the assembly module, but in that case, there will not be any screening for prokaryotic contigs. One drawback of this approach is that each eukaryotic/viral discovery pipeline has specific input data formatting requirements. This integration with non-prokaryotic pipelines, along with support for long reads, are priority features to be added to future major versions of Metaphor.

## Availability of Source Code and Requirements

Project name: Metaphor

Project homepage: https://github.com/vinisalazar/metaphor

Documentation: https://metaphor-workflow.readthedocs.io/

Operating system(s): Linux, Mac OS (Intel)

Programming language: Snakemake (Python 3)

Other requirements: Conda, Snakemake v7 or higher, Python 3.7 or higher.

License: MIT

RRID number: SCR_023701

## Supplementary Material

giad055_GIGA-D-23-00067_Original_Submission

giad055_GIGA-D-23-00067_Revision_1

giad055_GIGA-D-23-00067_Revision_2

giad055_GIGA-D-23-00067_Revision_3

giad055_Response_to_Reviewer_Comments_Original_Submission

giad055_Response_to_Reviewer_Comments_Revision_1

giad055_Response_to_Reviewer_Comments_Revision_2

giad055_Reviewer_1_Report_Original_Submission Thomas BrÃ¼ls -- 5/8/2023 Reviewed

giad055_Reviewer_2_Report_Original_Submission Po-Yu Liu -- 5/15/2023 Reviewed

giad055_Supplemental_Files

## Data Availability

This work uses data from the CAMI II challenge, available from [53–55]. Analysis code used in this article is available from [56]. Snapshots of our code and other data further supporting this work are openly available from the GigaDB repository [57].

## Additional Files

**Supplementary Fig. S1**. Differences between assembly strategies across datasets. Same data as Fig. 5, but including outliers.

**Supplementary Fig. S2**. Boxplot of bin scores across different strategies. Each data point is a genome bin, and the y-axis depicts bin scores from 0 to 1. Columns separate datasets, and colors represent different strategies. Numbers underneath each bar show the number of data points for that bar. Bin sets were dereplicated with dRep.

**Supplementary Fig. S3**. Boxplot of bin scores across different strategies for non-dereplicated data. Same as Fig. S2, but with non-dereplicated data. Each data point is a genome bin, and the y-axis depicts bin scores from 0 to 1. Columns separate datasets, and colors represent different strategies. Numbers underneath each bar show the number of data points for that bar.

**Supplementary Fig. S4**. Cumulative bin score and number of bins between binning strategies for the Marine and Strain Madness datasets. Solid lines show the same data as Fig. 6, and dashed lines show data based on bins prior to dereplication with dRep.

**Supplementary Table S1**. Summary of genome bins recovered from the Strain Madness dataset, CACB strategy. “Bin ID” indicates the binning algorithm that generated the bin, “Bin score *S_b_*” is the relative bin score, SCG refers to single-copy gene in “SCG completeness,” and “SCG redundancy,” “FastANI reference,” and “GTDB classification” refer to the reference genome and corresponding taxonomy assignment. Taxonomy determined with GTDB-Tk v2.3.0, reference data r214 [44].

## Abbreviations

CACB: coassembly, cobinning; CAMI II: Second Critical Assessment for Metagenome Interpretation; GRM: genome-resolved metagenomics; MAG: metagenome-assembled genome; NCBI: The National Center for Biotechnology Information; QC: quality control; SACB: single assembly, cobinning SASB: single assembly, single binning; SCG: single-copy marker gene.
