# Supplementary figures and images for: Metaphor—A workflow for streamlined assembly and binning of metagenomes
Source: Gigascience. 2023 Jul 31;12:giad055. doi: 10.1093/gigascience/giad055 (PMC10388702; doi:10.1093/gigascience/giad055)

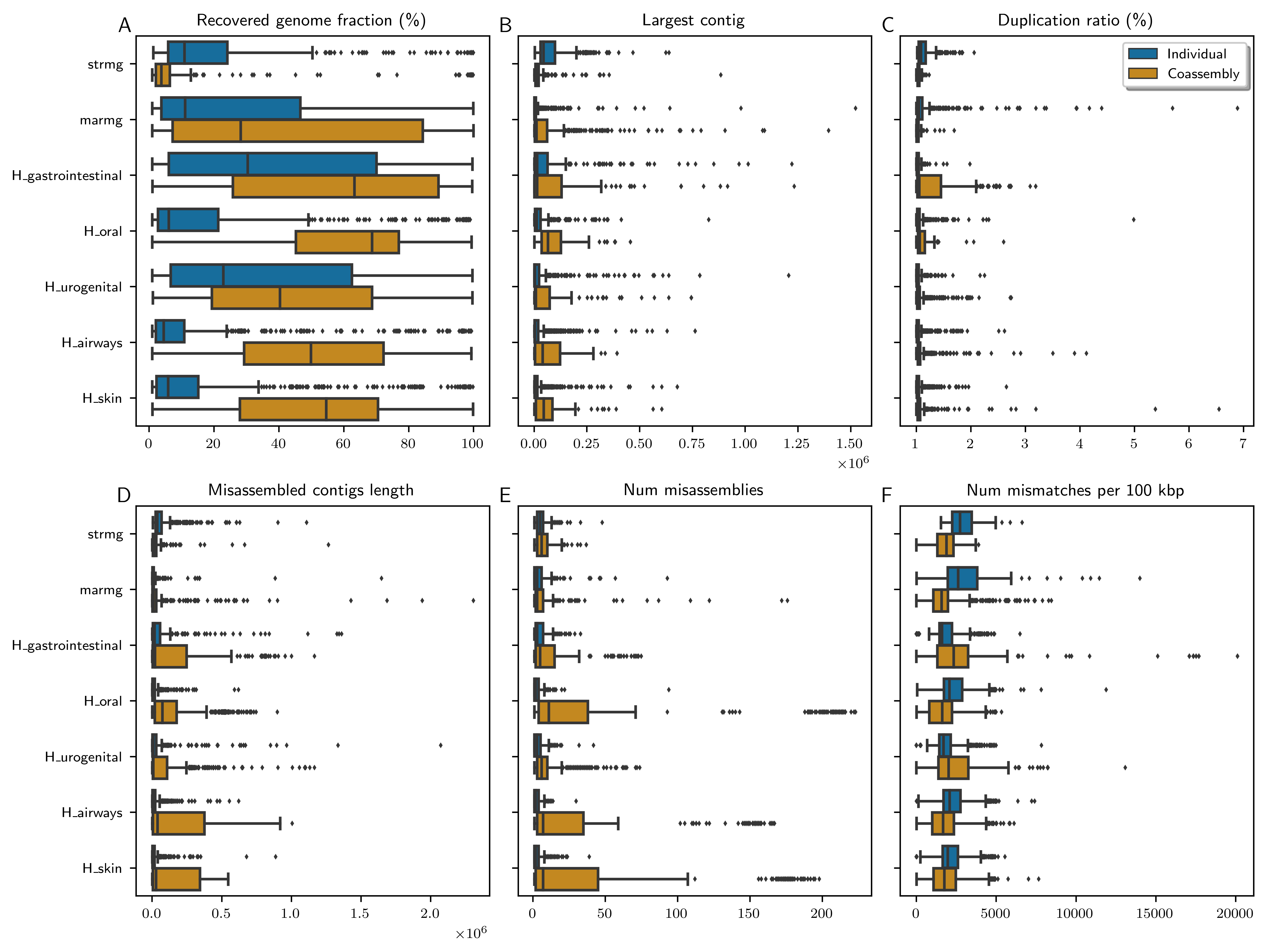

Supplement: giad055_Supplemental_Files [file giad055_supplemental_files.zip › FigS1_Supplementary Material.png]

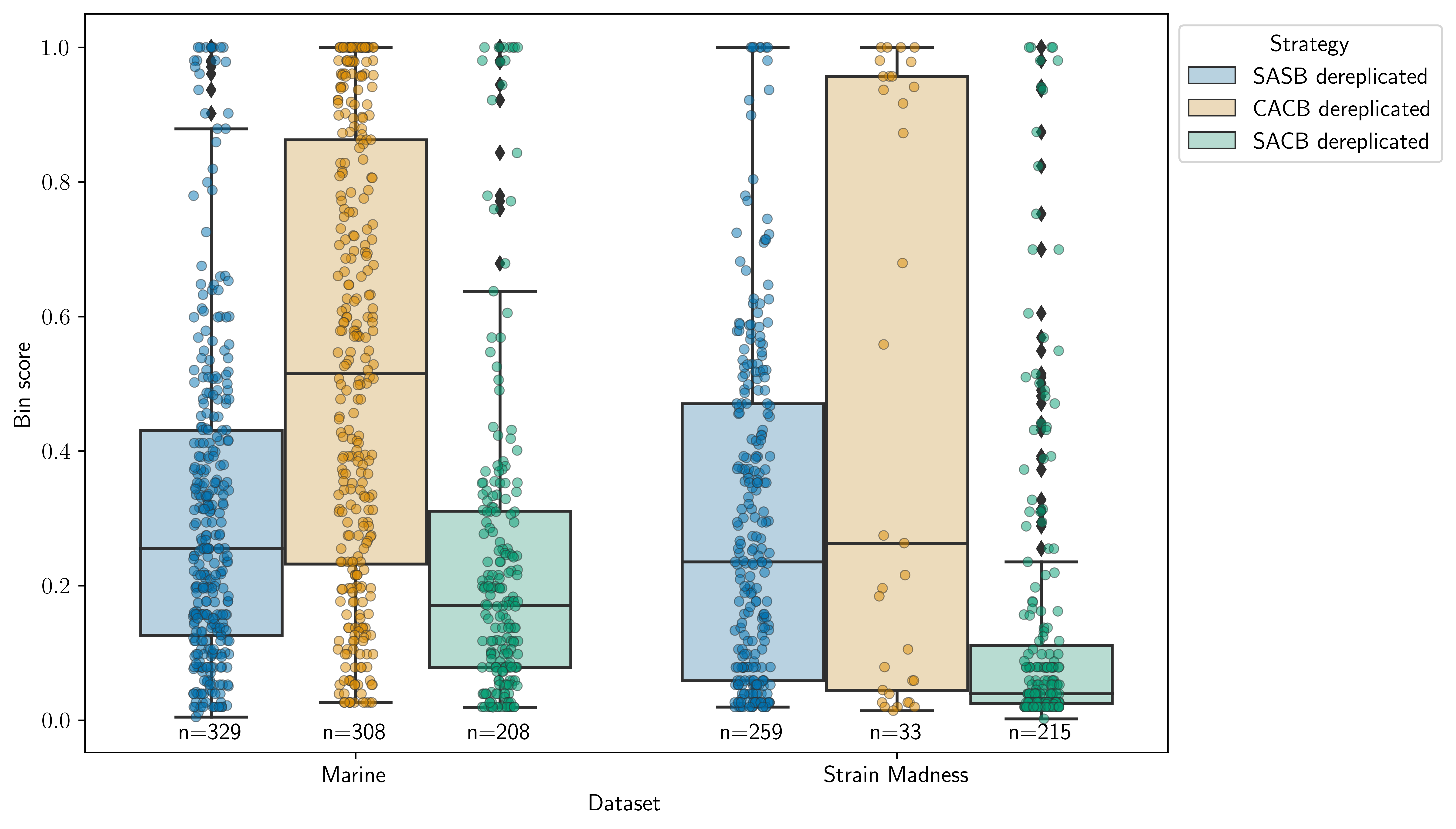

Supplement: giad055_Supplemental_Files [file giad055_supplemental_files.zip › FigS2_Supplementary Material.png]

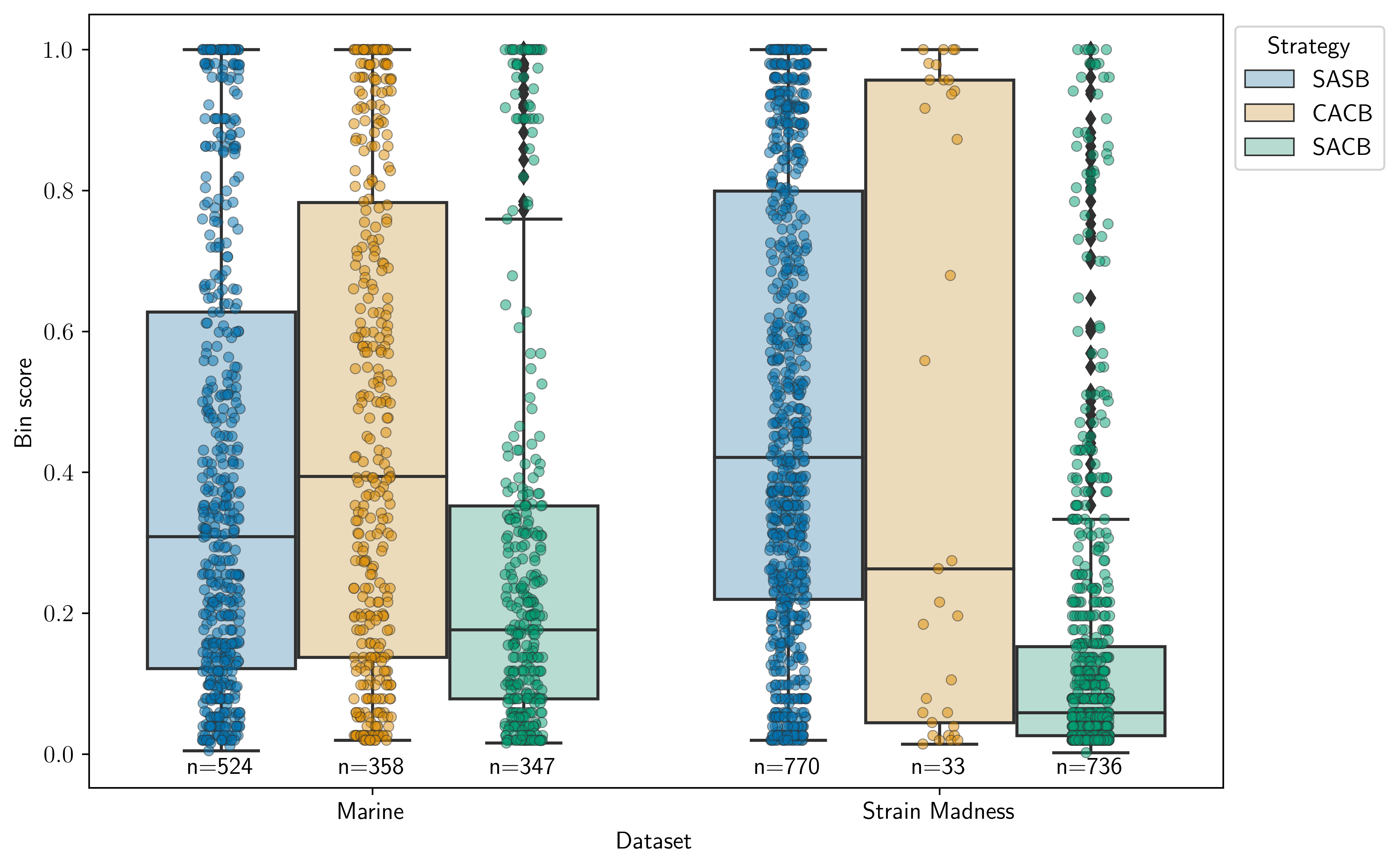

Supplement: giad055_Supplemental_Files [file giad055_supplemental_files.zip › FigS3_Supplementary Material.png]

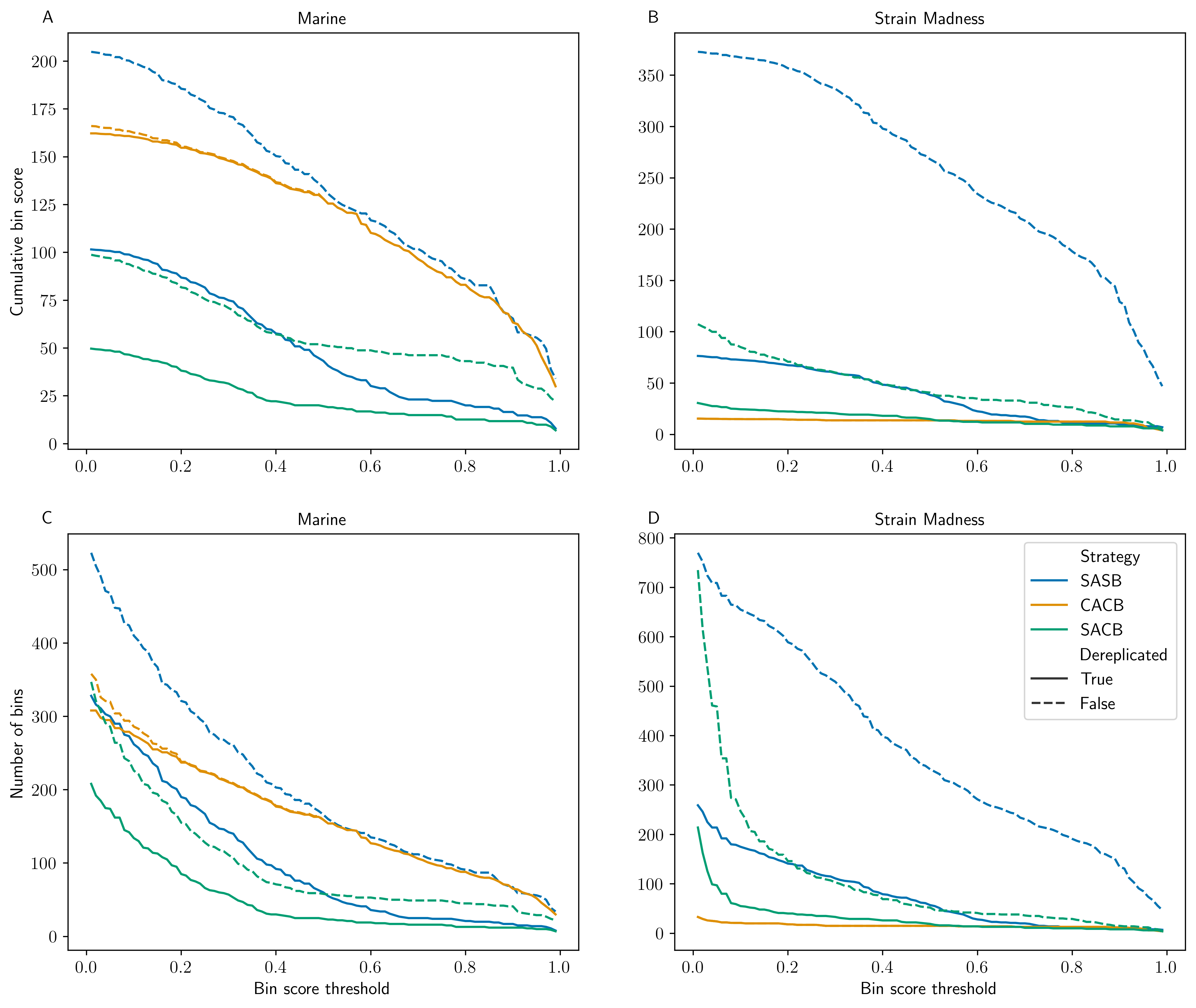

Supplement: giad055_Supplemental_Files [file giad055_supplemental_files.zip › FigS4_Supplementary Material.png]
