# Supplementary material for: Metaphor—A workflow for streamlined assembly and binning of metagenomes
Source: Gigascience. 2023 Jul 31;12:giad055. doi: 10.1093/gigascience/giad055 (PMC10388702; doi:10.1093/gigascience/giad055)
Supplement: giad055_Response_to_Reviewer_Comments_Original_Submission [file giad055_response_to_reviewer_comments_original_submission.pdf]

Author's Response To Reviewer Comments

Close

Dear editor,
See attached files in the submission

Close
