# Supplementary material for: Metaphor—A workflow for streamlined assembly and binning of metagenomes
Source: Gigascience. 2023 Jul 31;12:giad055. doi: 10.1093/gigascience/giad055 (PMC10388702; doi:10.1093/gigascience/giad055)
Supplement: giad055_Reviewer_1_Report_Original_Submission — Thomas BrÃ¼ls -- 5/8/2023 Reviewed [file giad055_reviewer_1_report_original_submission.pdf]

Reviewer Report

Title: Metaphor - A workflow for streamlined assembly and binning of metagenomes

Version: Original Submission **Date:** 5/8/2023

Reviewer name: Thomas BrÃ¼ls

Reviewer Comments to Author:

the authors present a snakemake-based workflow to automate and chain the main computational ingredients (assembly and binning) of genome-centric metagenomics; the authors developed a technically sound tool for this purpose, and by itself it is certainly valuable to the research community and worth of publication. however, even if the article is casted as a technical note -hence with an emphasis on the design, implementation and assessment of the tool-, I feel that a more thorough discussion of both its abilities and inabilities (e.g. strain resolution, detection of low abundance organisms, identification of virus bins, etc) would be worth for a more general audience. On the same token, a more deep discussion of some of the results obtained with their tool (see below) would be of interest and would also illustrate useful use cases.I would suggest the following modifications/additions:-the experiments with the strain madness dataset suggest that the genomes (or fragments thereof, i.e. the bins) resolved should be viewed as "species" genomes, or composite genomes possibly originating from multiple strains. if so, do the authors think this represents a hard limit to the assembly + binning approach, or could further existing tools (e.g. performing variant detection on top of cross-assembly before the binning step) be integrated or developed in the future for strain-resolution (i.e. to identify strains not dominant in any sample)?-related, a simple summary of the number of individual strains recovered in individual bins for the strain madness experiment would be interesting.-another issue that would be worth discussing in my opinion is the impact of genome abundance on the recovery of corresponding bins and their quality. the platform developed by the authors appears to be well suited for such kind of analyses and the results would be of both theoretical and practical interest. to put it simply, what is the minimal initial coverage of genomes required in order for them to be recovered in bins of a given size and quality?-rem: theses two issues (strain-level diversity and individual strain genome abundances) likely interact to limit bin resolution, and this could be mentioned by the authors.-the data presented by the authors suggest that the metabat binning engine significantly outperforms the other two tools (concoct and vamb, which are both widely used), see e.g Figure 2; what would account for that, and do the authors think this is a general observation (i.e. beyond the specific CACB setting or marine metagenome shown in Fig 2)?-a bin refinement step (based on the DAS tool and dereplication) is frequently mentioned but should be more detailed (including a precise definition of the bin quality metric used).further rather minor comments:-in the abstract, when mentioning "technical challenges associated with...", it would be worth mentioning that algorithmic challenges are present as well.-in the introduction, "It is hypothesised that pooled assembly and binning may lead to improved results when analysing communities with high genetic diversity, and to poorer results when there is a high level of intraspecies/strain-level diversity". I would assume there are many instances in the real world that are both, i.e. that present both high inter-species and intra-species

genetic diversity, what then?-in the future directions, the authors mention the identification of eukaryotic and viral contigs and bins, and could shortly elaborate how this could be done properly.-the sentence "In summary, our assessment of ..." at the end of the ms appears to have a syntactic problem.

Level of Interest

Please indicate how interesting you found the manuscript: Choose an item.

Quality of Written English

Please indicate the quality of language in the manuscript: Choose an item.

Declaration of Competing Interests

Please complete a declaration of competing interests, considering the following questions:

- Have you in the past five years received reimbursements, fees, funding, or salary from an organisation that may in any way gain or lose financially from the publication of this manuscript, either now or in the future?
- Do you hold any stocks or shares in an organisation that may in any way gain or lose financially from the publication of this manuscript, either now or in the future?
- Do you hold or are you currently applying for any patents relating to the content of the manuscript?
- Have you received reimbursements, fees, funding, or salary from an organization that holds or has applied for patents relating to the content of the manuscript?
- Do you have any other financial competing interests?
- Do you have any non-financial competing interests in relation to this paper?

If you can answer no to all of the above, write 'I declare that I have no competing interests' below. If your reply is yes to any, please give details below.

no

I agree to the open peer review policy of the journal. I understand that my name will be included on my report to the authors and, if the manuscript is accepted for publication, my named report including any attachments I upload will be posted on the website along with the authors' responses. I agree for my report to be made available under an Open Access Creative Commons CC-BY license (http://creativecommons.org/licenses/by/4.0/). I understand that any comments which I do not wish to be included in my named report can be included as confidential comments to the editors, which will not be published.

Choose an item.

To further support our reviewers, we have joined with Publons, where you can gain additional credit to further highlight your hard work (see: https://publons.com/journal/530/gigascience). On publication of this paper, your review will be automatically added to Publons, you can then choose whether or not to claim your Publons credit. I understand this statement.

Yes Choose an item.
